# Mass online training of health care workers during COVID-19: approach, impact, and outcomes for over 10,000 health care providers

**DOI:** 10.1016/j.puhe.2024.05.006

**Published:** 2024-08

**Authors:** A. Latif, M. Zaki, H. Shahbaz, S.A. Hussain, A.A. Daudpota, B. Imtiaz, F. Asghar, M.M. Hassan, M.A. Asghar, M. Aqeel, M.F. Khan, R. Khan, F. Mahmood, S. Nawab, A. Sabeen, M. Sohaib, S.F. Sultan, M. Tariq, H. Thawer, N. Ali, M. Jawwad, K. Niazi, A.A. Noorali, S.K. Amin, H. Atiq, Z. Samad, A.H. Haider

**Affiliations:** a Department of Anaesthesiology, Aga Khan University, Karachi, Pakistan; b Department of Community Health Sciences, Aga Khan University, Karachi, Pakistan; c Office of the Dean, Medical College, Aga Khan University, Karachi, Pakistan; d Department of Medicine, Aga Khan University, Karachi, Pakistan; e Department of Anaesthesiology, Surgical ICU and Pain Management, Ruth KM Pfau Civil Hospital Karachi, Dow University of Health Sciences, Karachi Pakistan; f Thawer Physiotherapy Ontario, Canada; g Department of Oncology, Aga Khan University, Karachi, Pakistan; h Department of Continuing Medical Education, Aga Khan University, Karachi, Pakistan; i Provost Office, Aga Khan University, Karachi, Pakistan; j Institute of Global Health and Development, Aga Khan University, Pakistan; k Department of Surgery, Aga Khan University, Karachi, Pakistan

**Keywords:** Medical education, Online education, E-Learning, Massive online open courses, Critical care, Public health, Disaster management, COVID-19

## Abstract

**Objectives:**

COVID-19 revealed major shortfalls in healthcare workers (HCWs) trained in acute and critical care worldwide, especially in low-resource settings. We aimed to assess mass online courses’ efficacy in preparing HCWs to manage COVID-19 patients and to determine whether rapidly deployed e-learning can enhance their knowledge and confidence during a pandemic.

**Study design:**

Retrospective cohort study.

**Methods:**

This international retrospective cohort study, led by a large Academic Medical Centre (AMC), was conducted via YouTube and the AMC's online learning platform. From 2020 to 2021, multidisciplinary experts developed and deployed six online training courses based on the latest evidence-based management guidelines. Participants were selected through a voluntary sample following an electronic campaign. Training outcomes were assessed using pre-and post-test questionnaires, evaluation forms, and post-training assessment surveys. Kirkpatrick's Model guided training evaluation to measure self-reported knowledge, clinical skills, and confidence improvement. We also captured the number and type of COVID-19 patients managed by HCWs after the trainings.

**Results:**

Every 22.8 reach/impression and every 1.2 engagements led to a course registration. The 10,425 registrants (56.8% female, 43.1% male) represented 584 medical facilities across 154 cities. The largest segments of participants were students/interns (20.6%) and medical officers (13.4%). Of the 2169 registered participants in courses with tests, 66.9% completed post-tests.

Test scores from all courses increased from the initial baseline to subsequent improvement post-course. Participants completing post-training assessment surveys reported that the online courses improved their knowledge and clinical skills (83.5%) and confidence (89.4%). Respondents managed over 19,720 COVID-19 patients after attending the courses, with 47.7% patients being moderately/severely ill.

**Conclusions:**

Participants' confidence in handling COVID-19 patients is increased by rapidly deploying mass training to a substantial target population through digital tools. The findings present a virtual education and assessment model that can be leveraged for future global public health issues, and estimates for future electronic campaigns to target.

## Introduction

The emergent and unprecedented nature of the SARS-COV-2 pandemic (COVID-19) revealed a global deficit in healthcare infrastructure, notably insufficient human resources with adequate training.^1^ Multidisciplinary staff, including physicians, nurses, and allied health professionals, are integral to the effective functioning of hospitals, especially in intensive care units (ICUs). The availability of appropriately trained staff is associated with improvement in mortality and length of stay.^2^

The global burden of critical illness is reportedly greatest in low-and middle-income countries (LMICs). Burgeoning numbers of non-communicable diseases amplify this disparity.^3^ Historically, the availability of healthcare resources in LMICs has been a constant challenge, with quality of care and health outcomes disproportionately lower compared to high-income countries (HICs).^4^ Since the number of COVID-19 cases and the infection fatality ratio were substantially higher in LMICs than in HICs, COVID-19 increased the demand for healthcare services, associated human resources, infrastructure, and equipment in LMICs, aggravating the strain on already limited resources.^5^^,^^6^ For example, a World Health Organization (WHO) study found that only 15 healthcare workers (HCWs) per 10,000 people were available in Pakistan.^7^ Other sources estimated that fewer than half of the required physicians and less than 5% of nurses were available in Pakistan.^8^ The COVID-19 pandemic acutely highlighted these shortfalls, necessitating strategies to train enough healthcare providers. However, the spread of COVID-19 and social distancing guidelines hampered training efforts.

Medical training initiatives arose globally to minimize the staffing deficits discussed above.^9^^,^^10^ As the pandemic progressed, knowledge of COVID-19 patient management techniques frequently changed and expanded. However, the time available to impart this knowledge remained limited.

Time constraints, lack of training opportunities, and new social distancing guidelines hindered in-person medical educational opportunities. Many institutions leveraged technology to deliver safely distanced educational interventions rapidly and repeatedly. Technology also decreased the time from inception to implementation of medical knowledge.^11^ This paradigm shift promoted pedagogical novelties such as e-learning, facilitating the global transition from traditional training methods to an entirely online exercise. Multiple studies have reported a positive impact on medical knowledge through e-learning curricula.^12^ E-learning provided a relatively inexpensive alternative to time-consuming and resource-intensive infrastructure upscaling.^13^^,^^14^ Recent studies have shown that e-learning could improve the quality of medical education in LMICs and provide contextually apt medical training.^15^^,^^16^ Furthermore, the increasing popularity of digital solutions, with over 110 million internet users in Pakistan reported in December 2021, suggested that e-learning interventions would be a feasible approach.

The WHO publicly praised Pakistan for its rapid and robust response to the spread of COVID-19.^17^ One of the initiatives taken by Pakistan to decrease the effects of the pandemic included the design and delivery of online education to increase the cadre of HCWs trained to manage COVID-19 patients (https://www.aku.edu/tele-icu/Pages/about.aspx). This initiative was led by one of the largest Academic Medical Centres in South Asia, the Aga Khan University (AKU) Pakistan. This retrospective study tracks the effectiveness of one of AKU's pandemic response strategies, which incorporated technology to rapidly deliver a series of online courses in an organized manner. These courses trained the existing healthcare workforce in managing COVID-19 patients.

### Hypothesis

Rapidly deployed online educational interventions will increase the knowledge acquisition of healthcare providers during the COVID-19 pandemic.

## Methods

**Fig. 1:**
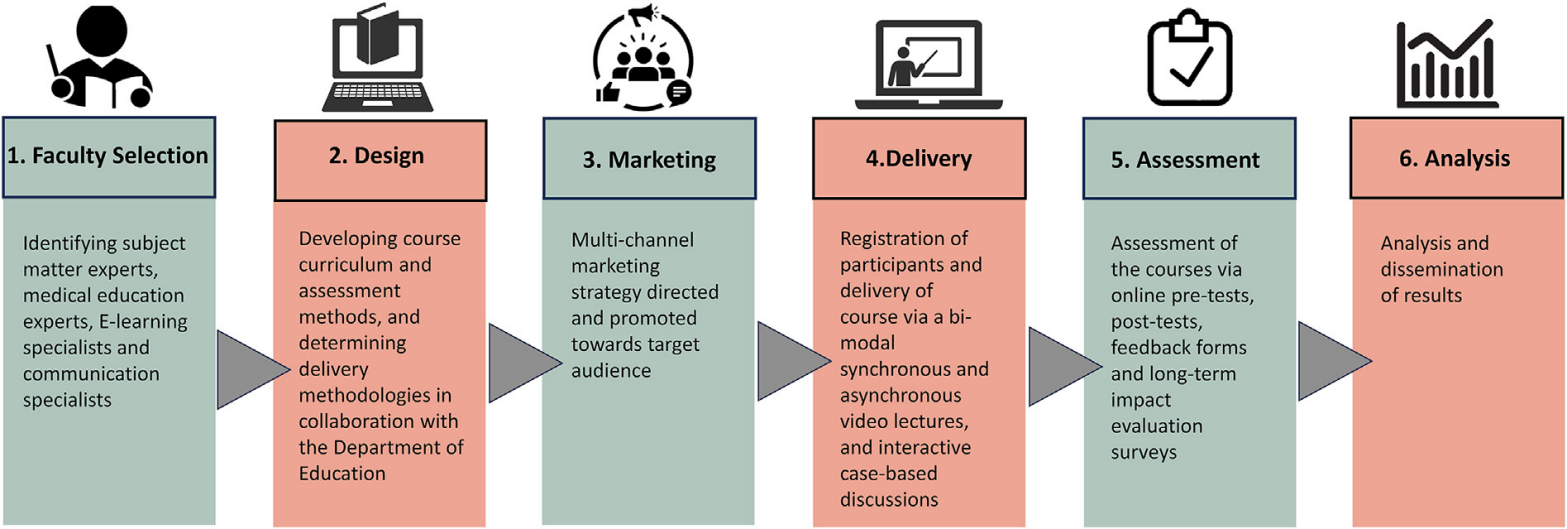
Process of the development and delivery of the online educational interventions.

Participants were offered six online free-of-cost training courses based on the latest evidence-based guidelines. The course development process is outlined in Fig. 1. The objectives, mode of delivery, and date(s) of each course are shown in Supplementary File 1.

### Study setting

This cohort study was led by the Aga Khan University's Medical College, accredited by the Accreditation Council for Continuing Medical Education (ACCME). The study was conducted on online platforms, including YouTube and the AKU's Virtual Learning Environment.

### Communication strategy for the intervention

A multi-tiered approach promoted the courses to the widest possible audience and disseminated vital medical information. Promotional materials (i.e., flyers) for each course shared course objectives, dates, and registration details. We exhausted multiple electronic mediums to increase the intervention's reach. E-flyers were distributed via emails, Facebook, Twitter, the university's official webpage (www.aku.edu), peer influence (peer-to-peer recommendations), and stakeholder public relations activities on television.

### Course design

We assembled a multidisciplinary team of subject matter experts from our institution as well as public and private sector academic medical institutes around the country to design each course. These experts determined the target audience, course objectives and content, mode of delivery, and evaluation methodologies. Faculty delivered these courses and created presentations, case studies, and pre-and post-test questionnaires. AKU's Department of Educational Development finalized all course objectives, content, and assessments (Supplementary File 1). All course completions were eligible for Continuing Medical Education activities certified by the American Medical Association, Physician's recognition Award (CME AMA PRA level 1 credit).

### Participants

Each course had an intended audience based on the theme of the content. There were no specific inclusion criteria; participants of all ages, designations, specialties, institutions, and locations could register for these courses.

The following terms categorized the study population:

1. Registrants: registered for the course/s and provided their demographic details
2. Participants: completed the pre-and post-tests for the course and were eligible for CME credits
3. Respondents: provided feedback after course completion

### Languages

Courses were conducted in English, as this is the official language of instruction for higher education in Pakistan. All lecturers were native speakers of both English and Urdu. Therefore, the course content was delivered in a discretionary combination of both languages to appeal to the broadest possible audience. The *Critical Care Course for Paramedics* was conducted solely in Urdu due to the target audience's perceived lack of comfort with English.

### Evaluation method

We assessed the effects of these interventions based on the four levels of Kirkpatrick's Model for evaluation of training (i.e., (1) Reaction, (2) Learning, (3) Behavior, (4) Outcomes).^18^ This model has been used extensively to evaluate medical education interventions and e-learning.^19^ Course assessments and evaluations measured the various levels of this model. These included pre- and post-tests, evaluation surveys disseminated immediately after course completion, and post-training assessment surveys disseminated one month after the course (Supplementary File 1).

### Data processing and statistical analysis

Pre-tests were conducted prior to the commencement of the course, and post-tests were disseminated at the conclusion of the training prior to certificate distribution. Results were compared using paired t-tests (for normally distributed data) and Wilcoxon-signed rank tests (for non-parametric data). We conducted an associative analysis to test the impact of (1) prior medical training (designation), (2) medical specialty, and (3) location of practice for the pre-and post-test results. We used one-way ANOVA (F-test) to compare normally distributed data and Kruskal–Wallis tests to compare non-parametric data. We used descriptive statistics to analyze the data from the evaluation surveys. All statistical analyses used a significance level of *P* < 0.05. We used STATA version 17 (Stata Corp, Texas) software and Microsoft Excel for the analysis.

### Role of the funding source

The study sponsor did not have any role in the study design, collection, analysis, and interpretation of data; writing of the report; or in the decision to submit the paper for publication.

## Results

A total of 10,425 HCWs registered for these online courses. The majority of the registrations were for the April 2020 *COVINAR* course (*n* = 4159, 39.9%). Most registrants for the courses were female (*n* = 5926, 56.8%). The registrants represented 154 cities in 38 countries worldwide, including 79 cities within Pakistan. Most registrants were from Pakistan (*n* = 8949, 97.2%). Occupation-wise, the largest group consisted of medical students/interns (*n* = 2143, 20.6%). A total of 584 medical institutions were represented, with the largest group being publicly owned institutes (*n* = 343, 58.7%) as shown in Table 1. Post-tests were completed by 1450 people (66.9%) out of the 2169 participants who registered in courses that had tests (Table 2).

**Table 1:** Demographic details of online course registrants.

	COVINAR (*N* = 4159)	Critical Care Course (*N* = 2294)	Prone Positioning for COVID-19 (*N* = 834)	Critical Care Course for Paramedics (*N* = 890)	COVINAR 2.0 (*N* = 1152)	Covid Management Webinar (*N* = 1126)	Total^a^ (*N* = 10,425)
**Sex**							
Male	1747	1118	294	358	399	580	4496
Female	2398	1167	537	529	749	546	5926
Not specified	14	9	2	3	3	0	3
**Designation**							
Administrative services	66	1	13	16	26	0	122
Academic Faculty	172	77	100	21	102	16	488
Non-academic physicians	386	535	104	56	98	26	1205
-Dentist	46	24	3	6	12	0	307
Fellow/resident	289	461	70	25	95	33	973
Medical Officer	729	415	29	43	135	43	1394
Nurse	377	256	57	315	63	56	1124
Pharmacist	141	42	13	25	44	0	265
Physiotherapist	53	28	128	30	14	3	256
Researcher	65	5	10	12	19	0	111
Respiratory therapist	4	22	2	20	2	0	50
Student/Intern	1017	231	231	191	444	29	2143
Technician	78	46	22	101	28	16	291
Non-medical professionals	48	0	36	26	19	15	144
Not specified	688	151	15	3	155	0	1552
**No. of Cities Represented**	76	85	31	61	55	44	154
**No. of Medical Institutions Represented**	346	267	92	112	158	246	584
**Type of Institution**							
Charity/Trust	31	27	9	12	10	13	64
Public	188	155	47	57	90	144	279
Private	126	83	34	42	58	88	235
Semi-private	1	2	2	1	0	1	6
**Marketing Metrics**							
Number of Posts/Tweets	42	25	0	6	0	2	78
Reach/Impressions	184,446	33,752	0	9466	0	9915	237,579
Engagement	7336	3651	0	828	0	673	12,488
Shares	450	245	0	11	0	19	725
Retweets	72	110	0	13	0	10	205
**Assessments**							
Prompt Evaluation Form Attempts	1626	540	275	269	568	310	3588
Pre- and Post-tests Attempts	N.A.	693	131	162	464	N.A.	1450
Post-training Assessment Survey Attempts	1254	475	157	126	353	142	2507

a The “Total” column has been considered with duplicates removed.

**Table 2:** Test results obtained from the individual online courses.

Course title	*n*	Pre-test score^a^	Post-test score^a^	Mean difference^b^ (95% CI)	*P*-value
Critical Care Course for Paramedics	182	71.3 ± 10.4	72.2 ± 11.6	0.91 (−0.33–2.13)	0.152
Proning Positioning for COVID-19	141	49.6 ± 21.0	54.5 ± 22.3	4.89 (1.89–7.91)	0.002
Critical Care Course	658	43.7 ± 12.7	48.3 ± 14.7	4.60 (3.75–5.47)	<0.001
COVINAR 2					
1st Day	369	26.7 ± 31.9	50.1 ± 34.1	23.44 (18.37–28.52)	<0.001
2nd Day	196	40.8 ± 27.9	46.1 ± 24.1	5.27 (0.35–10.19)	0.036
3rd Day	395	21.3 ± 28.1	54.2 ± 31.7	32.91 (28.75–37.07)	<0.001
4th Day	150	32.2 ± 19.8	38.0 ± 11.6	5.77 (2.00–9.55)	0.003

CI, confidence interval.

a Values for pre- and post-test scores are presented as mean ± SD.

b Mean difference is presented as post–pre-test score.

The AKU Medical College sent over 1000 emails to participants of previous training sessions to recruit them to participate in the study. The AKU's official Medical College Facebook page also made 49 posts, and the Dean of the AKU Medical College's Twitter account made 26 posts. These social media posts consisted of flyers containing course details. The terms ‘reach,’ ‘impressions,’ and ‘engagement’ are media analytics metrics frequently used within the context of social media. This social media strategy resulted in a reach/impression of 2,37,579 and a combined engagement of 12,488. Other users shared Facebook posts 725 times, and 205 retweets were made.

Results from the post-training assessment surveys found that 46% of respondents discovered the course (s) on the university's webpage (*n* = 1134), 29% found them on social media (*n* = 702), and 20% (*n* = 494) received recommendations from their peers (Supplementary File 1).

Post-tests were completed by 2091 out of the 5170 participants who registered for the *Critical Care Course*, *Critical Care Course of Paramedics*, *Prone Positioning for COVID-19*, *and COVINAR 2*.0 courses. The largest difference between pre-and post-test results was observed on the third day of the *COVINAR 2.0* course (32.91%, *P* < 0.001) as shown in Table 2. We found no association between pre and post-test results and participants' characteristics such as designation, institution, and city (Supplementary File 1).

**Fig. 2:**
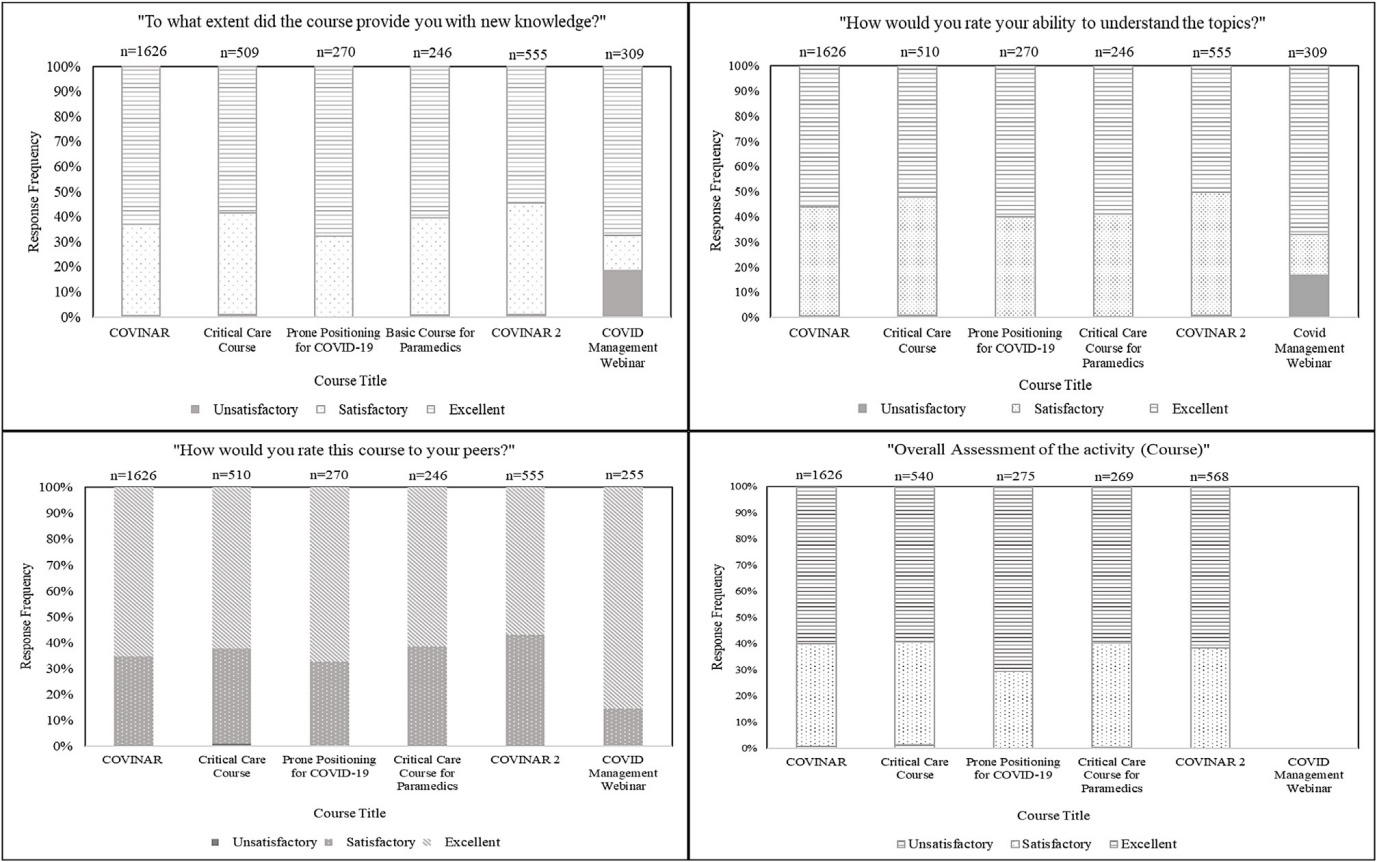
Participant perceptions as found in the post-training assessment surveys.

Evaluation surveys distributed promptly after course completion were completed by 3515 participants. The *Prone Positioning for COVID-19* course had the highest rating for providing participants with new knowledge (rated ‘excellent’ by 67.8% of respondents). Participants rated the topics presented in the *COVID Management Webinar* as the most understandable (rated ‘excellent’ by 67.1% of respondents); participants would give this course the highest recommendation to their peers (rated ‘excellent’ by 71.8% of respondents) (Fig. 2).

We evaluated the impact of the e-learning interventions with post-training assessment surveys. These assessment surveys were attempted by 2378 participants. An overwhelming majority of 83.5% (*n* = 1985) participants felt they could use the knowledge and skills gained from the courses in their practice, and 89.4% (*n* = 2377) reported increased confidence while managing patients. A Net Promoter Score (NPS) calculation assessed the degree to which participants would recommend these online interventions to their peers.^20^ There was a 16.1-point increase when comparing the NPS calculated from the evaluation forms distributed promptly after the courses (NPS = 29.1) versus that calculated from the post-training assessment surveys (NPS = 45.2). Please refer to Supplementary File 1.

**Fig. 3:**
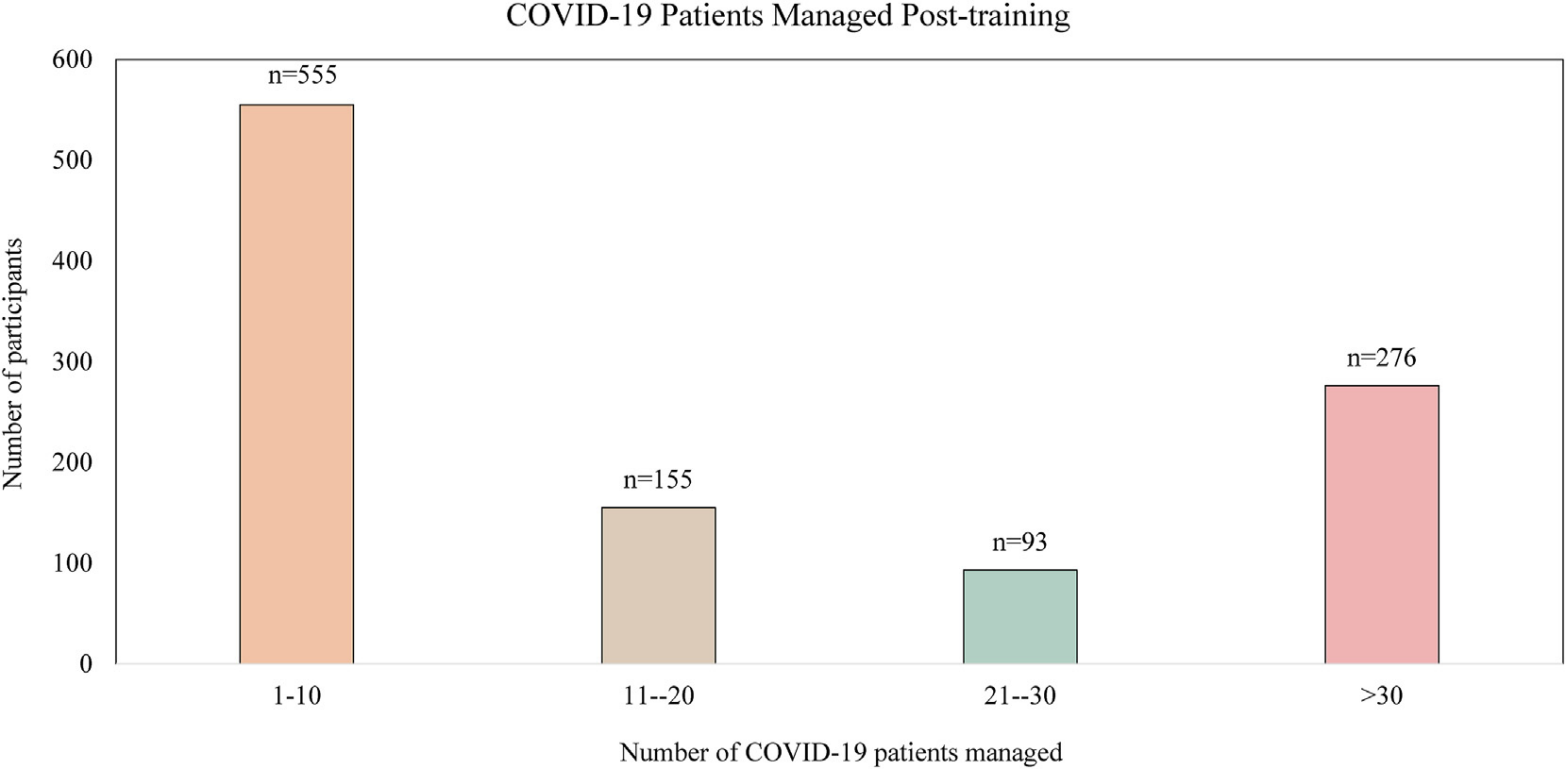
Number of COVID-19 patients managed by the participants of the e-learning interventions at Aga Khan University.

Some 45.5% (*n* = 1079) of respondents said they managed COVID-19 patients after attending these courses. Respondents reportedly managed over 19,720 COVID-19 patients post-training, of which 47.7% (*n* = 9406) were moderately or severely ill (Fig. 3, Supplementary File 1). When extrapolating the total number of participants who completed our online courses, the potential impact of these educational interventions is augmenting care for 43,223 patients, with a potential ability by our course registrants to reach over 190,000 COVID-19 patients.

## Discussion

As the world scrambled to address the disastrous effects of the COVID-19 pandemic, Pakistan's COVID-19 response was internationally praised by organizations such as the WHO and Centre for Global Development, attributing the country's success to its well-developed guidelines and use of medical training interventions.^21^^,^^22^ Interventions such as ours were integral to this response. By designing and deploying six online courses, the AKU, in conjunction with the Government of Pakistan, imparted critical COVID-related knowledge to over 10,000 participants, who then provided better care and potentially improved outcomes for COVID-19 patients. These e-learning courses provided a comprehensive curriculum with global reach, increased the knowledge and confidence of HCWs in managing COVID-19 patients, and considerably impacted patient management.

The development and rapid deployment of a multi-channel marketing strategy assisted in the global mass training of participants, which was the need of the hour during the COVID-19 pandemic. Ours was a first-of-its-kind educational intervention to reach such a large audience, particularly during a pandemic. Most participants learned about the courses via information uploaded on AKU's official website. The official website is a valuable tool for our communications team and a trusted source of information for potential participants; therefore, it proved an effective promotional tool. Given the reputation of AKU, offerings are frequently picked up by other institutions across the region, helping account for the extent of our audience. Additionally, our diverse faculty from different facilities and sectors of the healthcare system undoubtedly helped spread word of offerings in their own circles. Considerations for the future include partnerships with other AMCs and ministries of health to promote educational offerings more directly to specific target audiences in terms of practitioners, specialties, or locales of interest. In our case, although the target audience for these courses was HCWs in critical care settings, registrations came from participants in various medical specialties and non-medical backgrounds thus indicating that the curriculum delivered was of interest to a diverse audience. This could be due to the redeployment of HCWs from other specialties into ICUs to counter staff shortages, the burgeoning interest in COVID-19, the user-friendliness of these courses, and the dearth of COVID-related information available elsewhere, particularly during the initial stages of the pandemic.^20^^,^^23^, ^24^, ^25^ It is important to note that medical students represented the single largest group of participants, probably due to a combination of their “availability” when everything shut down during the early days of the pandemic to develop protocols, their comfort with online learning as the youngest members of the healthcare workforce, and their desire to contribute to the collective challenge of a global pandemic.

Our communication and promotions strategy resulted in a ground-breaking number of registrations. To achieve 10,425 registrations and 2365 course completions, we had to reach 237,579 people using our promotion strategy. Comparing the number of registrants to the social media reach/impressions shows that 1 in every 22.8 reach/impressions led to a registration, and 1 in every 1.2 engagements led to a registration. Further, 1 in every 66.2 reach/impressions and 1 in every 3.5 engagements resulted in post-test completion, respectively. Traditionally, academicians and the agencies that fund their work consider registrations and course completions as benchmarks for measuring the success of educational interventions. However, we have gone a step further and quantified the relationship between communication strategies and reach related to the registrations and the subsequent course completions achieved. This knowledge can be used by future educators and operations management teams to develop robust communication strategies to enhance the scope of their interventions. Mass online courses have the advantage of reaching a large and diverse audience, including healthcare professionals from different regions and backgrounds. Because of the virtual nature of the intervention, we were able to train participants from over 150 cities. LMICs have fragile health care systems with inadequate resources to train HCWs, especially those in remote areas. Virtual courses are more cost-effective than traditional in-person training programs, as they eliminate the need for travel and physical facilities. E-learning offers flexibility in terms of when and where healthcare workers can access training materials. This is especially important during a pandemic when HCWs may have irregular schedules or need to quarantine.

Just as it is necessary to capture a wide audience, obtaining participant feedback and reviews is pertinent for assessing the courses' quality and identifying improvement areas. Level 1 of Kirkpatrick's Model details the importance of evaluating participants' reactions to determine the overall effect of the educational interventions. The feedback from evaluation forms distributed promptly after course completion shows that the participants gave our online courses an overall ‘excellent’ rating. The *COVID Management Webinar* received the highest rating (71.8%). Additionally, more than 50% of the participants felt that these courses provided them with new knowledge and that the topics presented were understandable. This signifies that, at large, participants were satisfied with the courses, fulfilling the requirements of this level of Kirkpatrick's Model. Further, feedback received from participants post-course completion reflects on their overall satisfaction, as the majority expressed that they would recommend these courses to their peers. The post-training assessment survey results give an NPS of 45.2, suggesting that participants found the courses beneficial. This represents a 55.3% increase from the NPS calculated directly after course completion, indicating that participants found the knowledge gained from the online interventions to be more beneficial after time had elapsed since completion, potentially owing to its application in their clinical duties.

Leveraging technology to facilitate the dissemination of medical education programs is an alluring concept, particularly given the shortage of trained healthcare professionals. However, previous courses did not provide a globally available medical curriculum or cover the specific intricacies of managing COVID-19 patients.^26^ A study conducted in 2013 hypothesized that global threats, including pandemics, could motivate the application of technology for e-learning initiatives.^27^ In addition to a shortage of healthcare personnel, insufficient training and skill development pose significant challenges for HCWs in developing nations. A systematic review assessing the optimum strategies for training HCWs in LMICs concluded e-learning to be an effective training method.^14^ Our study shows that remote training using e-learning initiatives was feasible and effective, with test results for all courses in this series showing an initial paucity in clinical knowledge followed by an enhancement of knowledge as shown in the pre-and post-test results, respectively. More specifically, results attained after completing the third day of the *COVINAR 2.0* course exhibited an impressive 32.91% (*P* < 0.001) increase in test scores. This satisfies frameworks like Kirkpatrick's, showing that knowledge has been acquired from our e-learning interventions. The lack of association between training (i.e., medical, nursing, and allied health training) and test results in the courses targeting all HCWs suggests that the information provided in these courses was helpful to participants regardless of the type of medical background. This indicates that this online education series ameliorated the baseline knowledge of HCWs and that such an e-learning framework can be designed to be effective.

Prerequisites for managing critically ill patients are access to the correct knowledge and the confidence needed to manage such patients.^28^ The third level of Kirkpatrick's Model emphasizes the importance of behavioral changes due to education. We assessed the behavioral impact of these courses via evaluation forms administered promptly after the conclusion of each course and post-training assessment surveys distributed at least 30 days after course completion. These surveys revealed that participants felt more confident in their knowledge and skills after completing the courses. Previous studies have also reported that e-learning initiatives prompted positive behavioral changes in their participants.^29^ Through e-learning, HCWs can be provided with up-to-date information on the latest research, guidelines, and best practices. Our findings show that HCWs have widely embraced online education. These results establish a propitious outlook for the future implementation of medical education delivery via e-learning modalities.

Lastly, and most importantly, we assessed the potential impact of these e-learning initiatives using post-training assessment surveys. Evaluation of the clinical impact of educational interventions is the highest and most pivotal level of Kirkpatrick's Model. However, measuring this outcome is a reportedly difficult task.^18^ To our knowledge, this is one of the few studies that attempt to link online medical education with clinical impact. While estimating the direct incremental impact on patient outcomes is difficult, the improvement in knowledge and very positive participant evaluations and feedback suggest that patient care and outcomes might have improved. Of note, the only course that did not demonstrate a significant knowledge benefit was the one targeting paramedics. This group is often derived from a different socioeconomic and educational background in LMICs like Pakistan, which can have an influence on how comfortable they are with online learning and platforms, as well as their access to internet connectivity. Further study is needed as this did not manifest in our other courses, possibly due to the fact that paramedics were a relatively small proportion of the participant population. Importantly, our assessments indicate that participants found opportunities to translate their knowledge into bedside clinical practices, and felt that it improved the care of their patients. Close to half of those that responded after 30 days managed about 20,000 COVID-19 patients, providing a coarse estimate for the proportion of the relevant target population of HCW's and patients that such an approach can reach. Dedicated comparative controlled trials in this area are needed to quantify the impact of online mass educational approaches on patient outcomes.

Although e-learning is a growing field in educational delivery, significant challenges are associated with this pedagogical novelty. The most apparent challenge related to e-learning is the occurrence of participant attrition.^30^ Our study shows that the completion rates of the post-tests and evaluation surveys were considerably lower than the number of registrations. Current literature suggests that participant attrition is not limited to any level of education or designation.^30^ However, this is particularly evident in the medical community as HCWs have busy schedules often characterized as ‘unsocial working hours.’^31^ This instantiates the lack of time available to participate in professional development training. Strategies to overcome these barriers to e-learning should be conceptualized in future studies.

### Conclusions

The COVID-19 pandemic revealed a shortage of trained healthcare providers worldwide. To address this shortage, we rapidly developed and deployed a series of online educational interventions to upskill HCWs for managing COVID-19 patients. Our findings prove that such online training can successfully build the capacity of skilled HCWs in LMICs like Pakistan and globally. The results from this study demonstrated that online education had a substantial impact on the knowledge acquisition and patient management of thousands of HCWs. This presents an optimistic outlook for the future implementation of medical education delivery via e-learning modalities and suggests that these interventions can be used as prototypes to develop future online courses in case of pandemic preparedness.

## Author statements

### Acknowledgements

We would like to thank the many faculty and staff who assisted in the design and delivery of the courses.

### Ethical approval

This study was reviewed and approved by the Ethics Review Committee of the Aga Khan University (ERC ID: 2020-4869-10701).

### Funding

10.13039/100000865Bill and Melinda Gates Foundation, USA (INV 017820).

### Competing interests

The authors declare that they have no competing interests.

### Authors' contributions

All authors have made substantial contributions in this work. AL, ZS, AH, MT, NA, BI were involved in the conceptualization and design of the study. MZ, HS, SAH, AAD, BI, FA, MMH, MAA, MA, MFK, RK, FM, SN, AS, MS, SFS, MT, HT, NA, MJ, KN, SKA, HA, ZS, AH, AL were involved in the execution of the study. MZ, HS, SAH, AAD, FA, MMH, AAN, MJ, KN, SKA, HA, ZS, AH, AL were involved in the data collection, analysis, and interpretation. MZ, HS, SAH, HA, ZS, AH, AL were involved in the development of the first draft and incorporation of feedback from the team. All authors had full access to all the data in the study, participated in the final review and editing of the manuscript and accept responsibility to submit it for publication.

### Consent for publication

Not applicable.

### Consent to participate

Participant data was obtained on a voluntary basis with clear statement of the lack of impact on any outcome.

### Data sharing

We are committed to data transparency. All data generated or analysed during this study are included in this published article [and its supplementary information files]. The de-identified datasets used and/or analysed during the current study are available from the corresponding author on reasonable request.
