# Supplementary material for: Mass online training of health care workers during COVID-19: approach, impact, and outcomes for over 10,000 health care providers
Source: Public Health. 2024 Aug;233:193–200. doi: 10.1016/j.puhe.2024.05.006 (PMC11283886; doi:10.1016/j.puhe.2024.05.006)
Supplement: Multimedia component 1 [file mmc1.pdf]

Contents

COVINAR: An update on the COVID-19 Pandemic.......... 2

Caring for Covid-19 Patients in Intensive Care Areas; A two-day basic course for paramedical staff.......... 3-3

COVINAR - 2: An Update on Treatment and Management of COVID-19 Patients.......... 4-4

Critical Care Management of COVID-19: Webinar.......... 5-5

Pre- and post-course test assessment results…………..7-15

Course content and objectives

Course Name	*Objectives*	*Course Content*	*Mode of Delivery*	*Assessment Method*	*CME hours*	*Date Offered*
**COVINAR: An update on the COVID-19 Pandemic**	• To update knowledge on evidence-based research on COVID-19 pandemic<br>• To understand the management of mild, moderate and severe COVID-19 infection<br>• To understand the concept of donning and doffing<br>• To learn the association of COVID-19 cardiovascular diseases and cancer<br>• To understand the effects of COVID-19 pandemic on mental health	<u>Day 1:</u><br>• Protecting Healthcare Providers in the wake of COVID-19<br>• Clinical Manifestations & Diagnosis of COVID-19<br>• Corona Viruses: Epidemiology, Pathogenesis and Diagnosis<br><u>Day 2:</u><br>• Challenges of outpatient screening and tele-medicine<br>• Challenges of triaging COVID-19 patients in Emergency Department<br>• Challenges of managing COVID-19 patients in the wards<br><u>Day 3:</u><br>• COVID-19 treatment options: What is the evidence?<br>• Infection prevention & control guidelines for dealing with COVID-19<br><u>Day 4:</u><br>• Ventilator management in COVID-19 patients<br>• Practicing social distancing and prevention in COVID-19<br>• Challenges of quality assurance in a COVID-19 hospital<br><u>Day 5:</u><br>• Zoonosis & One-health: SARS-CoV2 in the historic and geographic context<br>• From fear to resilience: Strategies for well-being in pandemic times<br>• Cancer patients’ management during the COVID-19 pandemic<br><u>Day 6:</u><br>• COVID-19 and cardiovascular complications<br>• Mental health challenges during COVID times<br>• COVID-19 and pregnancy<br>• Responding to COVID-19 Pandemic: Testing Resilience of Health Systems<br>• Closing remarks	Online synchronous lectures (Free Open Access Medical Education)	• Prompt feedback surveys<br>• Post-training evaluation survey	6·00 AMA PRA Category 1 Credit Hours	20-30th April 2020

Critical Care Course for COVID-19	• Develop fundamentals of critical care medicine<br>• Enable diagnosis and management of critically ill COVID-19 patients<br>• Provide evidence-based treatment (including ICU preparation to safely and appropriately manage cases)	Day 1:<br>• Introduction to ICU & Assessment of the Critically ill Patient (COVID-19 patient)<br>• Acute Respiratory Failure and special considerations in Covid-19<br>• Airway Management, Basics of ABG's & Radiological findings in COVID-19<br>Day 2:<br>• Basics of Mechanical Ventilation<br>• Sedation, Analgesia & Paralysis in ICU<br>• Sepsis, Shock & Appropriate use of antibiotics<br>Day 3:<br>• Infection Control in ICU & COVID-19 Patient<br>• ICU Management of COVID-19 Patient: Current EBM<br>• Skill stations/demonstrations/tutorials: Donning & Doffing for COVID-19<br>Day 4:<br>• Hemodynamic Assessment & Management in Critically Ill Patients with COVID-19<br>• Assessment of the Critically Ill Child<br>• Acute Respiratory Failure and Mechanical Ventilation in COVID-19 Child<br>• Skill stations/demonstrations/tutorials: Airway & CPR in COVID-19	• Pre reading<br>• Online synchronous interactive lectures<br>• Videos	• Pre-test<br>• Post-test<br>• Prompt feedback surveys<br>• Post-training evaluation survey	8·5 AMA PRA Category 1 Credit Hours	5-14th May 2020
**Caring for Covid-19 Patients in Intensive Care Areas; A two-day basic course for paramedical staff**	• Perform donning and doffing of personal protective equipment (PPE)<br>• Describe infection control measures required for providing safe care<br>• Provide basic physiotherapy, proning and positioning for ICU patients<br>• Delineate steps in administration of medication to ICU patients<br>• Explain the use of common ICU equipment	Day 1:<br>• Donning and doffing of PPE<br>• Medication safety and administration<br>• Use of common ICU equipment<br>• Basics of mechanical ventilation<br>• Demonstration of Ventilator basics and common ICU equipment<br>Day 2:<br>• General care of Covid-19 patients in ICU<br>• Physiotherapy of ICU patients, proning and positioning for Covid-19 patients<br>• Infection control practices in ICU<br>• Cleaning and disinfection of equipment	Online synchronous interactive lectures	• Pre-test<br>• Post-test<br>• Prompt feedback surveys<br>• Post-training evaluation survey	6·0 AMA PRA Category 1 Credit Hours	19th - 21st May 2020

	and basic steps of mechanical ventilation<br>• Provide general care to Covid-19 patients in ICU<br>• Detail the essential steps of cleaning equipment					
**Prone Positioning in Patients with COVID-19; From Theory to Practise**	• Understand the pathophysiology of acute respiratory failure and the measures to improve oxygenation<br>• Understand the current literature on the proning in mechanically ventilated and conscious patients<br>• Understand the indications and contraindications of proning and to learn to identify patients with ARDS due to COVID-19 that would most likely benefit from proning<br>• Acquire a preliminary understanding of the practical aspect of proning	• Pathophysiology of acute respiratory failure & measures to improve oxygenation<br>• Literature review of proning in mechanically ventilated patients and conscious patients<br>• Indications and contraindications of proning; who is most likely to benefit<br>• Introduction and Video - Demonstration of Proning	• Online synchronous live lectures<br>• Videos	• Pre-test<br>• Post-test<br>• Prompt feedback surveys<br>• Post-training evaluation survey	2·5 AMA PRA Category 1 Credit Hours	17th June 2020
**COVINAR - 2: An Update on Treatment and Management of COVID-19 Patients**	• Update knowledge on evidence-based research on COVID-19 pandemic<br>• Understand the management of mild, moderate and severe COVID-19 infection<br>• Understand the concept of donning and doffing<br>• Learn the association of COVID-19	<u>Day 1:</u><br>• COVID-19;.What have we learnt in 6 months?<br>• COVID-19 treatment options: Updated evidence<br>• Infection prevention control guidelines for COVID-19	Online synchronous interactive lectures	• Pre-tests<br>• Post-tests<br>• Prompt feedback surveys<br>• Post-training evaluation survey	5·0 AMA PRA Category 1 Credit Hours	7th-16th September 2020

	cardiovascular diseases and cancer	Day 2:<br>• Challenges of managing COVID-19 patients<br>• Gastrointestinal manifestations of COVID-19				
		Day 3:<br>• Respiratory Support in COVID Patients<br>• Pulmonary manifestations and long-term outcomes				
		Day 4:<br>• Updates in treatment of cancer patients with COVID<br>• Cardiovascular complications in COVID-19<br>• Responding to COVID 19 Pandemic:<br>• Testing Resilience of Health Systems				
**Critical Care Management of COVID-19: Webinar**	• Learn to diagnose and manage patients with COVID- 19<br>• Learn how proning helps in gas exchange in COVID patients<br>• Learn tips to interpret the arterial blood gases<br>• Learn to manage ventilation (Invasive vs	Day 1:<br>• Diagnosis & management of mild to moderate COVID-19 patients<br>• Management of critically ill COVID-19/ Cytokine response syndrome<br>• Role of Proning in COVID-19	Online synchronous interactive lectures	• Prompt feedback surveys<br>• Post-training evaluation survey	4·0 AMA PRA Category 1 Credit Hours	10th and 11th August 2021

	noninvasive) in COVID patients	Day 2:<br>• Interpretation of ABGs<br>• Respiratory failure and its management with NIV<br>• Invasive Ventilation in COVID-19				

Critical Care Course for Paramedics; Pre and Post-test Results

Variable	N	Mean (SD)	Median (IQR)	P-value
**Pre-test Score**	162	43·43 (6·37)	45 (39-48)	**0·253**
**Post-test Score**	162	43·96 (7·22)	45 (40-49)	

***Wilcoxon signed-rank test**

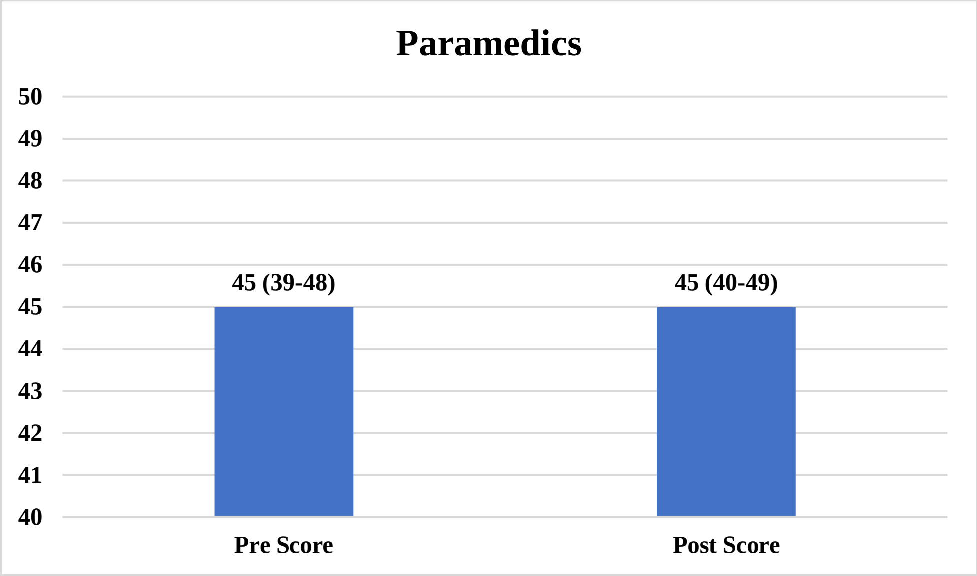

	Public Sector Hospital	Private Sector Hospital	p-value
	N=87	N=75	
Difference between pre and post-test score	0 (-4-5)	0 (-2-4)	0·310

***Kruskal-Wallis equality-of-populations rank test**

	Student/Intern	Consultant/Physician/M.O	Nurse	Physiotherapist	Other	p-value
	N=48	N=21	N=54	N=10	N=28	
Difference between pre-test and post-test Score	0 (-3-4·5)	0 (-3-5)	·5 (-2-4)	-·5 (-7-5)	0 (-3-3)	0·910

***Kruskal-Wallis equality-of-populations rank test**

	Sindh	Other	p-value
	N=138	N=24	
Difference between pre-test and post-test Score. Mean (SD)	0·79 (5·40)	-1·00 (4·97)	0·130

F-test

Prone Positioning for Covid-19 Pre and Post-test Results

**Proning Score**			
**Variable**	**Obs**	**Mean (SD)**	**p-value**
Pre-test Score	131	4·97 (2·1)	<0·001
Post-test Score	131	5·57 (2·25)	

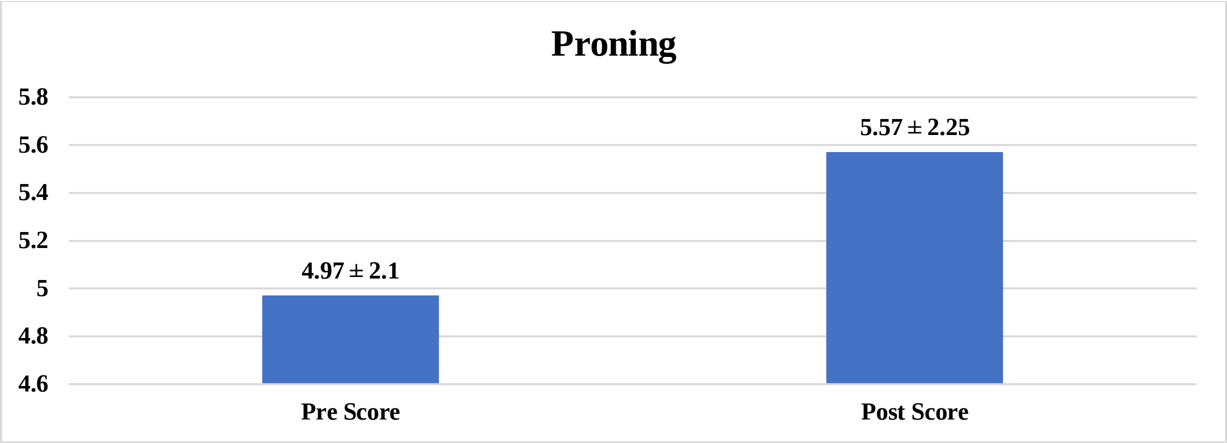

	Public Sector Hospital	Private Sector Hospital	
	N=32	N=79	p-value
Difference between pre-test and post-test Score. Mean (SD)	0·63 (2·32)	0·39 (1·64)	0·55

F-test

	Teaching staff	Fellow/Resident	Student/Intern	Consultant/Physician/M.O	Nurse	Physiotherapist	Other	p-value
	N=13	N=12	N=56	N=14	N=10	N=16	N=10	
Difference between pre-test and post-test Score. Mean (SD)	1·15 (1·07)	0.25 (1·29)	0·38 (2·20)	1·21 (1·63)	1·40 (1·78)	0·56 (1·21)	0·00 (1·83)	0·32

F-test

	Sindh	Other	p-value
	N=108	N=11	
Difference between pre-test and pos-test Score. Mean (SD)	0·49 (1·84)	0·64 (2·11)	0·81

F-test

Critical Care Course Pre and Post-test Results

Critical Care

Variable	N	Mean (SD)	Median (IQR)	P-value
**Pre-test Score**	693	13·06 (3·87)	13 (10-16)	**<0·001*******
**Post-test Score**	693	14·54 (4·39)	15 (11-17)	

***Wilcoxon signed-rank test**

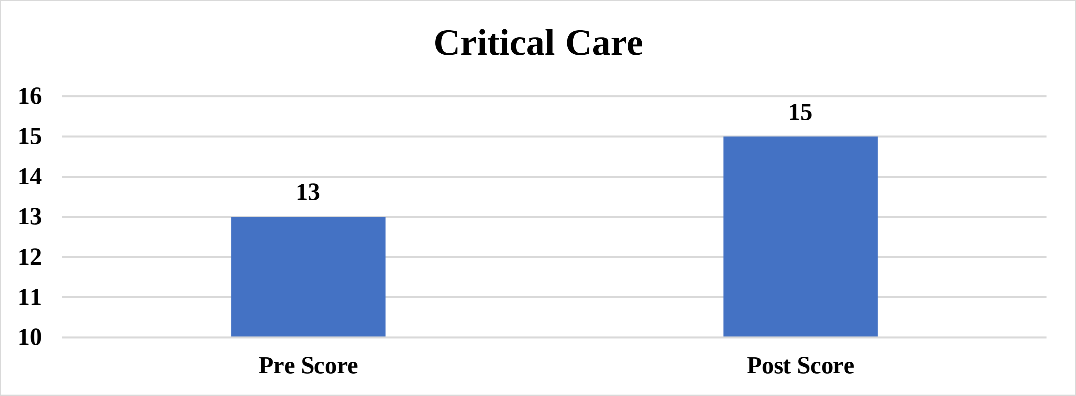

	Public Sector Hospital	Private Sector Hospital	p-value
	**N=385**	**N=303**	
Difference between pre-test and post-test Score. Mean (SD)	1 (-1-3)	1 (-1-3)	0·650

***Kruskal-Wallis equality-of-populations rank test**

	Teaching staff	Fellow/Resident	Student/Intern	Consultant/Physician/M.O.	Nurse	Other	p-value
	**N=53**	**N=154**	**N=104**	**N=261**	**N=67**	**N=51**	
Difference between pre-test and post-test Score. Mean (SD)	1 (-1-3)	2 (0-4)	1 (-1-3)	1 (0-3)	1 (-1-3)	1 (0-4)	0·100

***Kruskal-Wallis equality-of-populations rank test**

	KPK	Punjab	Sindh	ISL/Bal/Kashmir	International	p-value
	N=45	N=65	N=514	N=22	N=44	
Difference between pre-test and post-test Score. Mean (SD)	2 (0-3)	2 (-2-3)	1 (-1-4)	0 (-1-3)	2 (-1-4)	0·140

***Kruskal-Wallis equality-of-populations rank test**

COVINAR 2 Pre and Post-test Results

Covinar 2 day-1

Variable	N	Mean (SD)	Median (IQR)	P-value
**Pre-test Score**	464	0·54 (0·62)	0 (0-1)	**<0·001*****
**Post-test Score**	464	1·06 (1·12)	1 (1-1)	

***Wilcoxon signed-rank test**

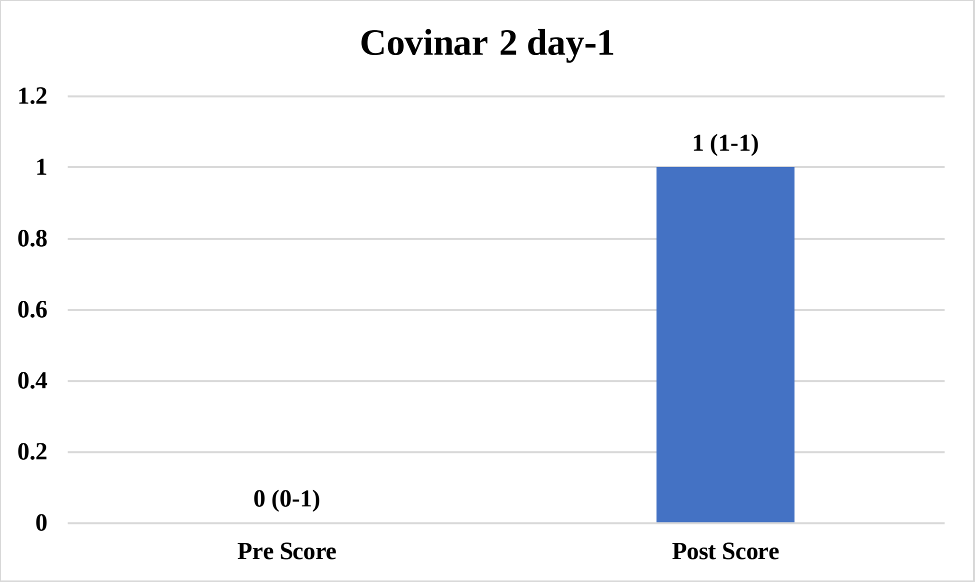

Covinar 2 day-2

Variable	N	Mean (SD)	Median (IQR)	P-value
**Pre-test Score**	197	1·18 (0·79)	1 (1-2)	**<0·003***
**Post-test Score**	197	1·39 (0·70)	1 (1-2)	

Paired t test

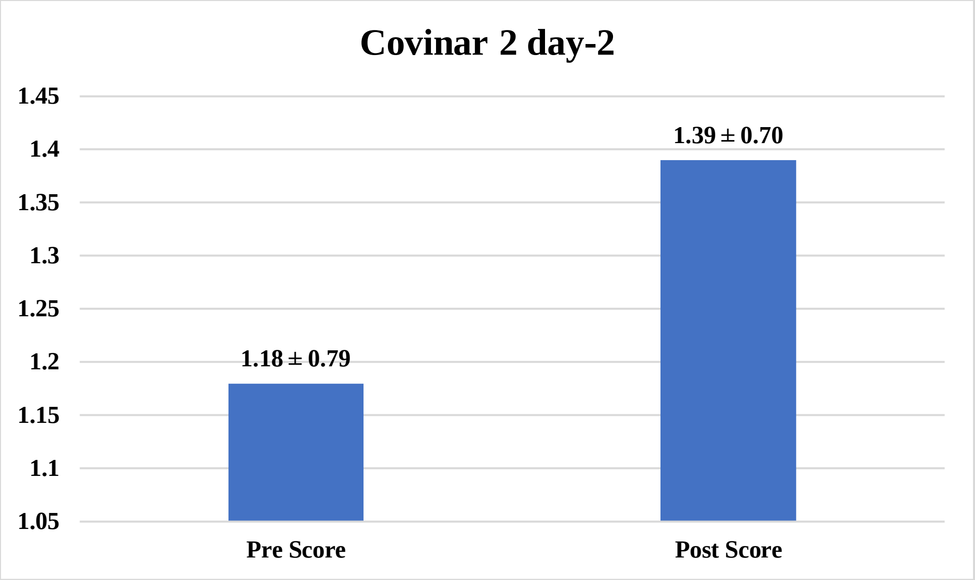

Covinar 2 day-3

Variable	N	Mean (SD)	Median (IQR)	P-value
**Pre-test Score**	418	0·43 (0·56)	0 (0-1)	**<0·001*****
**Post-test Score**	418	1·13 (1·16)	1 (1-1)	

***Wilcoxon signed-rank test**

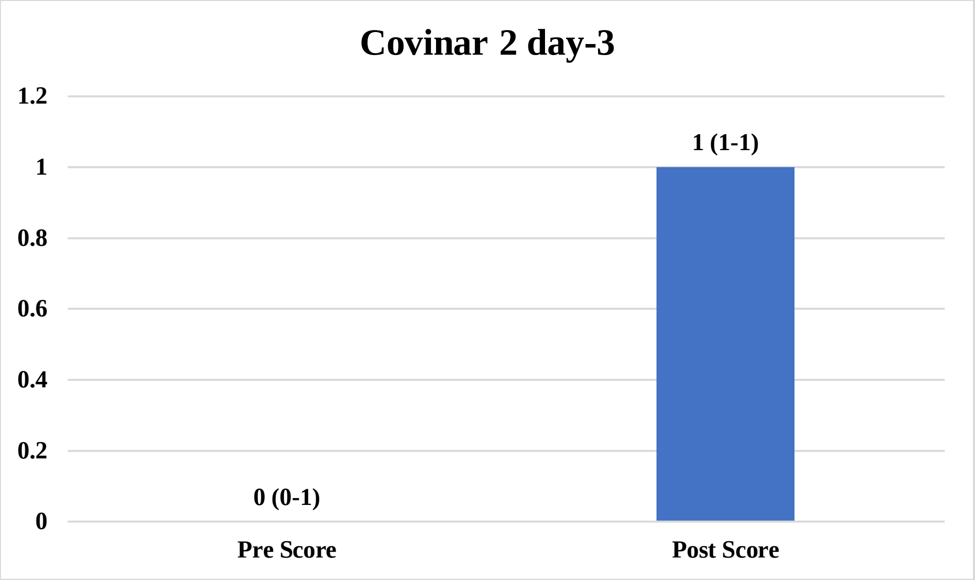

Covinar 2 day-4

Variable	N	Mean (SD)	Median (IQR)	P-value
**Pre-test Score**	300	0·94 (0·57)	1 (1-1)	**<0·001*****
**Post-test Score**	300	1·16 (0·37)	1 (1-1)	

***Wilcoxon signed-rank test**

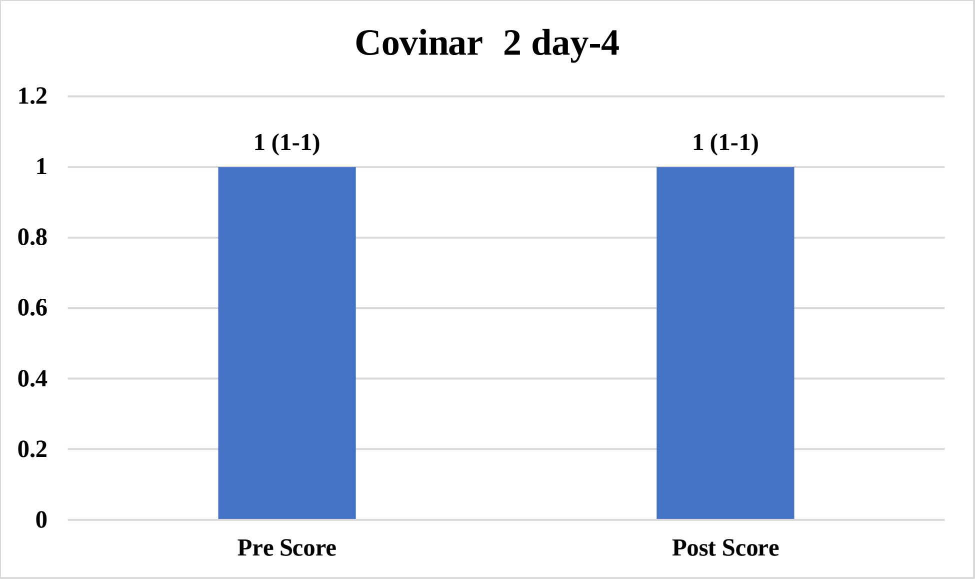
Covinar 2 day-4
1.2
1
0.8
0.6
0.4
0.2
0
1 (1-1)
1 (1-1)
Pre Score
Post Score
