# Supplementary material for: Mass online training of health care workers during COVID-19: approach, impact, and outcomes for over 10,000 health care providers
Source: Public Health. 2024 Aug;233:193–200. doi: 10.1016/j.puhe.2024.05.006 (PMC11283886; doi:10.1016/j.puhe.2024.05.006)
Supplement: Multimedia component 2 [file mmc2.pdf]

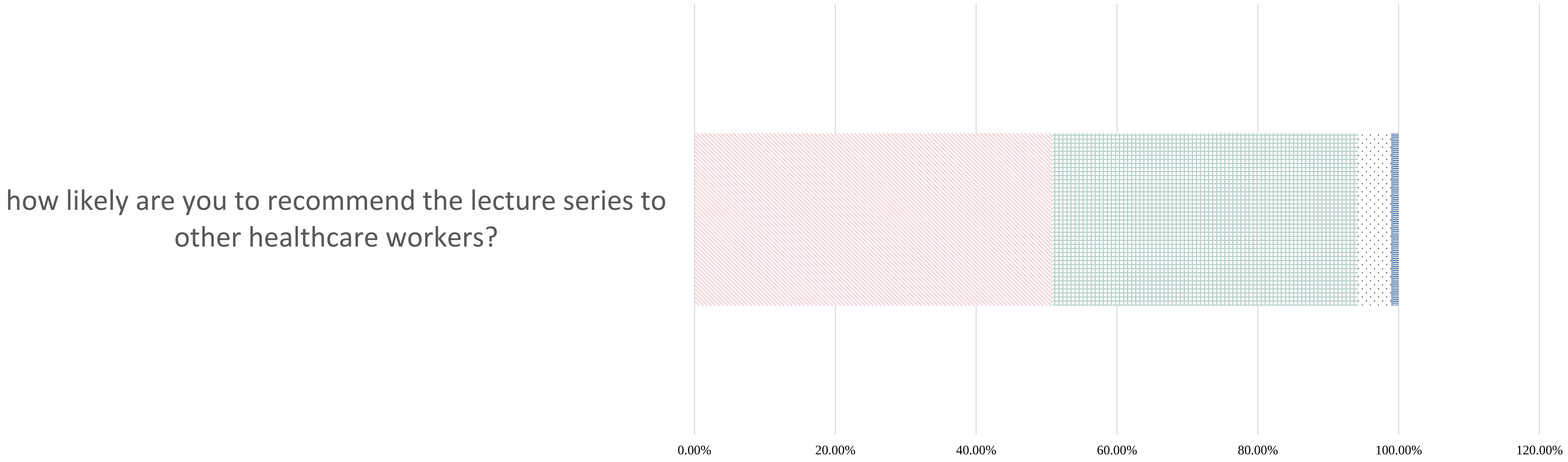

	how likely are you to recommend the lecture series to other healthcare workers?
Extremely likely	50.88%
Likely	43.40%
Neutral	4.67%
Unlikely	0.29%
Extemely unlikely	0.76%

This chart represents the participants' answers to "How likely are you to recommend the lecture series to other health care workers?", asked in the post-training evaluation survey. The data from participants of all courses has been combined for this analysis. NPS is calculated as values representing "extremely likely" (Promoters) less values representing "extremely unlikely" and "unlikely" (detractors).

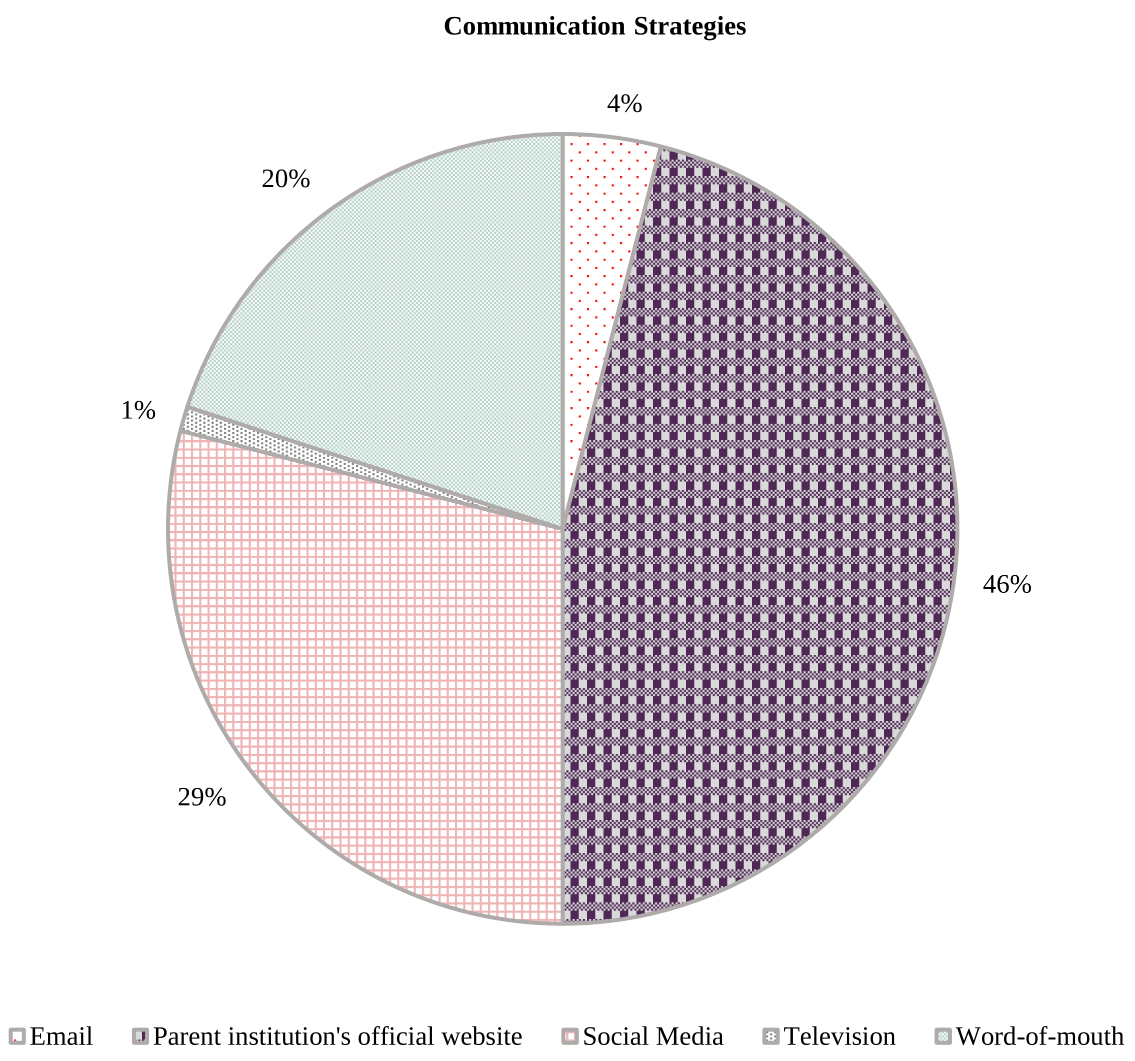

Reach of communication strategy to the participants

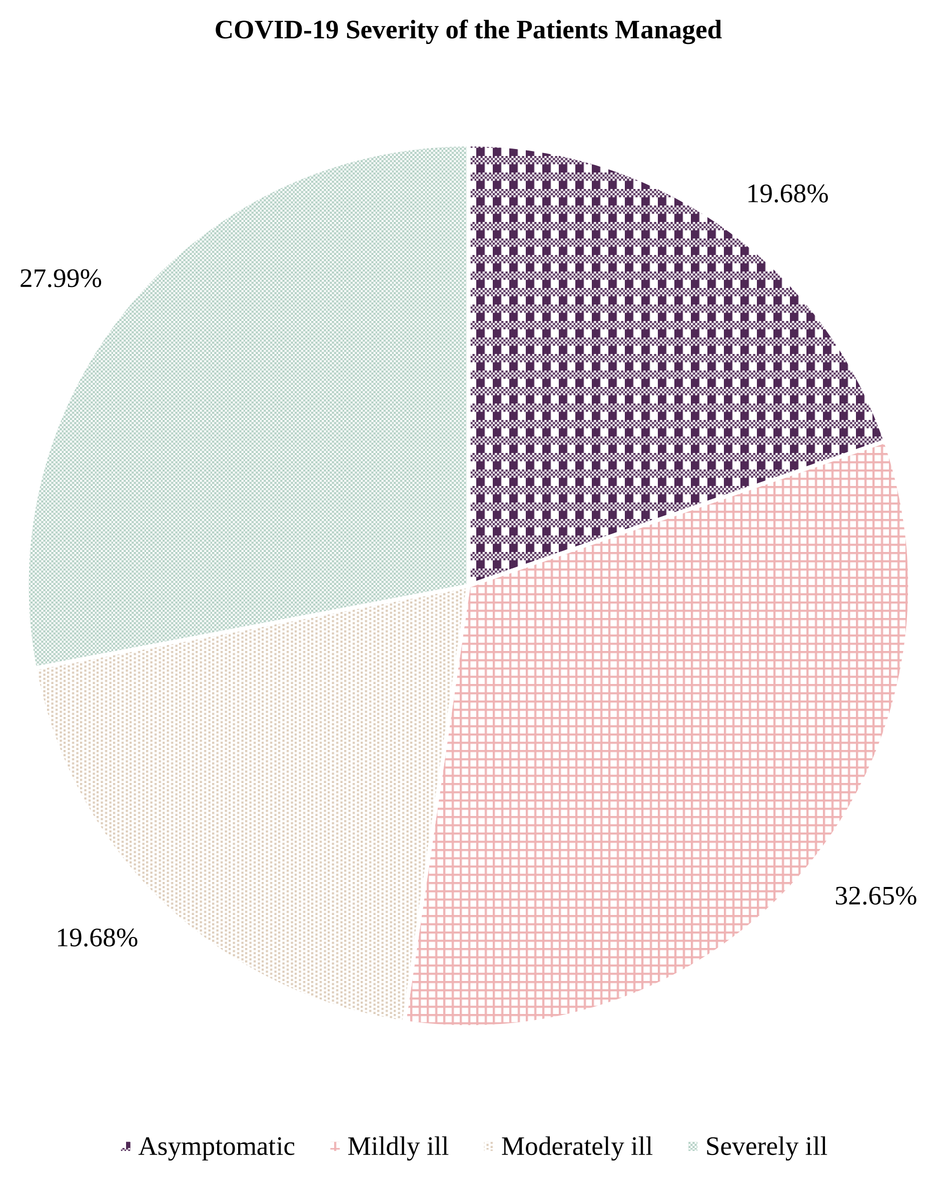

COVID-19 symptom severity of patients managed by the online educational intervention participants post-course completion
